# The Discourse Surrounding Polycystic Ovary Syndrome on TikTok: A Social Media Analysis

**DOI:** 10.3390/healthcare12222253

**Published:** 2024-11-12

**Authors:** Anna Horvath, Kendall Chaffin, Sophie Ahmad, Vidhani S. Goel, Dale M. Netski, Rooman Ahad, Kavita Batra, Rebecca Lee

**Affiliations:** 1 Kirk Kerkorian School of Medicine at UNLV, University of Nevada, Las Vegas, NV 89106, USA; horvaa1@unlv.nevada.edu (A.H.); chaffk2@unlv.nevada.edu (K.C.); ahmads1@unlv.nevada.edu (S.A.); 2 School of Public Health, University of Nevada, Las Vegas, Las Vegas, NV 89154, USA; vidhani.goel@unlv.edu; 3 Office of Faculty Affairs, Kirk Kerkorian School of Medicine at UNLV, University of Nevada, Las Vegas, NV 89102, USA; dale.netski@unlv.edu; 4 Department of Medical Education, Kirk Kerkorian School of Medicine at UNLV, University of Nevada, Las Vegas, NV 89106, USA; 5 Department of Pediatrics, Kirk Kerkorian School of Medicine at UNLV, University of Nevada, Las Vegas, NV 89102, USA; 6 Office of Research, Kirk Kerkorian School of Medicine at UNLV, University of Nevada, Las Vegas, NV 89102, USA; 7 Department of Gynecologic Surgery & Obstetrics, Kirk Kerkorian School of Medicine at UNLV, University of Nevada, Las Vegas, NV 89106, USA

**Keywords:** PCOS, TikTok, social media, misinformation, online content

## Abstract

Background/Objectives: Individuals are turning increasingly towards online resources, such as TikTok, to educate themselves on their medical conditions. Polycystic ovary syndrome (PCOS) is a prominent example, as women report dissatisfaction with the diagnosis process and treatment options. This study aims to provide a content analysis of the quality of PCOS health information on TikTok. Methods: A total of 325 TikTok videos were screened. Pertinent data, including likes, comments, shares, and views, along with video content concerning symptoms, interventions, and provider interactions were analyzed. Two reviewers independently used a modified DISCERN criteria to assess the quality of information for each video. A logistic regression was also utilized to model the probability of healthcare professionals creating educational videos. Results: A total of 238 videos met the eligibility criteria for analysis. Videos had a median of 468,400 views (Q1 = 146,400, Q3 = 1,100,000) and 18,000 likes (Q1 = 5631, Q3 = 65,100). The mean modified DISCERN scores were 3.6 ± 0.9 for physicians (n = 23), 2.0 ± 1.1 for non-physician healthcare providers (n = 52), and 1.0 ± 0.2 for non-healthcare professionals (n = 141) (*p* < 0.001). Healthcare professionals were 10.9 times more likely to create educational videos in comparison to non-healthcare professionals (*p* < 0.001). Conclusions: TikTok videos related to PCOS attract considerable engagement but provide low-quality information. Most videos were made by non-healthcare professionals, who discussed treatment options with limited or no research. Healthcare providers should be aware of the impacts on patients’ preconceived notions and help to improve patient education.

## 1. Introduction

Access to quality health education for adolescents and young adults is highly variable across the world [1]. Many individuals rely on social media to find health information, particularly for those with limited access to health resources [2,3,4]. The growing popularity of TikTok opens new frontiers for medicine [5]. It is becoming increasingly common to share personal experiences with diseases, treatment options, at-home remedies, and more, which can help foster a sense of community and educate the general user on various medical topics [5]. While TikTok can be a unique, effective way to reach a large audience, it may also allow misinformation to be disseminated rapidly [6]. With Tik Tok not being screened to the same degree as a peer-reviewed article, it is essential to better understand the content being disseminated [7]. This could be vital to educating providers about the preconceived notions their patients may have when visiting their offices.

Social media users typically fall between the ages of 18–29 years, with 81% of people ages 30–49 using at least one platform [8]. According to the Pew Research Center, women were more active than men on social media platforms and spent more time on short-form video content on social media [9,10]. Recently, TikTok has seen an increase in content related to women’s reproductive health, with one example being polycystic ovary syndrome (PCOS) [11]. PCOS is a common endocrinopathy, affecting 4–8% of reproductive women [12].

According to the American College of Obstetricians and Gynecologists (ACOG), PCOS is characterized by polycystic ovaries, hyperandrogenism, and ovulatory dysfunction [13]. Common manifestations of the condition include menstrual disorders, infertility, hirsutism, and weight changes [13]. PCOS increases the risk of type 2 diabetes and cardiovascular disease [13].

PCOS often garners significant attention on social media, where it is frequently depicted through a lens of personal anecdotes and trending wellness advice [11]. This heightened visibility can raise awareness and foster a sense of community but it can also perpetuate misconceptions. PCOS is influenced by various factors, including genetics and environmental conditions [14]. The manifestations of the disease and the effectiveness of treatments can differ widely giving conflicting information to social media users [14]. This can create frustration amongst users worried about misinformation as expressed through online platforms such as Reddit [15]. While individual users have expressed concern, there is a lack of peer-reviewed research diving into PCOS content on social media. This social media investigation aims to provide a descriptive content analysis of the quality of PCOS information on TikTok. Symptoms and treatments discussed in videos will be compared to current ACOG guidelines on management of PCOS. Additionally, videos will be examined to determine differences between video quality and engagement based on demographics and healthcare affiliation. The study aims to serve as a resource for healthcare providers to educate themselves to provide more informed care to their patients.

## 2. Materials and Methods

### 2.1. Selection of Videos

This study was deemed exempt by the University of Nevada, Las Vegas Institutional Review Board (UNLV-2022-169). A new TikTok account was created to collect videos for our study. A search was conducted on TikTok (https://www.tiktok.com) in April 2024 for the search term “PCOS”. The search results were organized according to the TikTok algorithm. The first 325 uniform resource locators (URLs) were collected and recorded on a Microsoft Excel spreadsheet.

The inclusion criteria were videos uploaded to TikTok from a public account that were relevant to the condition in question. The exclusion criteria were videos not in English and videos unrelated to PCOS. Two authors independently verified the video selection, and a third author resolved any disagreements.

### 2.2. Data Collection

Engagement metrics such as views, likes, comments, saves, shares, and followers were recorded for each video. The number of views per day (views divided by days since the video was posted), views per like (number of views divided by the number of likes), and likes per day (number of likes divided by days since the video was posted) were calculated. It was also recorded whether videos were sponsored by a product or company and whether TikTok verified the user’s account. Videos were then categorized by themes, including “Education/Informational”, “Humor/Entertainment”, “Testimonial/Seeking Advice”, or “Advertisement”. Sources (including physicians, non-physician healthcare providers, students, talk shows, for-profit companies, patients, and other individual users) were recorded for each video based on self-reported data from users. These categories were further stratified according to physicians, non-physician healthcare professionals, and non-healthcare professionals. Non-physician healthcare professionals included pharmacists, physician assistants, nurse practitioners, nurses, students studying healthcare, and dieticians. Non-healthcare professionals were defined as all other user qualifications or users who did not state their qualifications. The sex, race, and age of the person posting the video were perceived by the researchers analyzing these videos.

The characteristics discussed in the videos, including whether the user mentioned having PCOS, the stigma of PCOS, and/or an interaction with a provider, were recorded. All videos were viewed and assessed independently by two authors. Any disagreements were resolved through discussion between the two authors.

### 2.3. Content Analysis

Videos were analyzed for symptoms and interventions mentioned that related to PCOS. Possible symptoms included “weight gain”, “acne”, “hirsutism”, “infertility”, “cysts”, and “mental health”. Interventions included “diet”, “exercise”, “OCPs”, “non-OCP medications”, and “supplements”. A single post often referenced multiple topics. The symptoms and interventions were recorded if they were discussed within the video but excluded if they were only referenced in the caption. The symptoms and interventions were recorded and compared to the American College of Obstetricians and Gynecologists (ACOG) practice bulletin, which details common symptoms and well-researched interventions [13].

### 2.4. Video Reliability

Video reliability was assessed using a modified version of the DISCERN criteria consisting of 5 questions [16]. The DISCERN instrument was developed to judge the quality of consumer health information. The tool is utilized to determine whether a video is of high or low quality. Questions included: “Are the aims clear and achieved?” “Are reliable sources of information used?” “Is the information presented balanced and unbiased?” “Are additional sources of information listed for patient reference?” and “Are areas of uncertainty mentioned?” Each video was scored from 0 to 5 using the sum of the questions, with 0 indicating ‘low quality’ and 5 indicating ‘high quality’. All videos were analyzed independently by two authors.

### 2.5. Statistical Analysis

The unit of analysis was the TikTok video. First, a univariate analysis was conducted to describe the data in terms of the measures of central tendencies (e.g., mean, median), measures of dispersion (standard deviation), and range for the numeric variables. The categorical variables were presented as counts and proportions. The normal approximation to the binomial distribution method was used to calculate 95% confidence intervals of proportions in the univariate analyses. An independent-samples *t*-test (a type of bivariate test) was performed to determine if there were any statistically significant differences in the metrics of social media engagement (i.e., likes, views, and comments) and in the data validation scores (Modified DISCERN) among different groups of users, video types, and video sources. A logistic regression was also utilized to model the probability of healthcare professionals creating educational videos. Estimates for the parameters were obtained through the maximum likelihood estimation method with 95% Wald’s confidence limits for the logistic model. All analyses were conducted using SPSS version 27 and SAS 9.4.

## 3. Results

### 3.1. Descriptive Statistics

**Figure 1:**
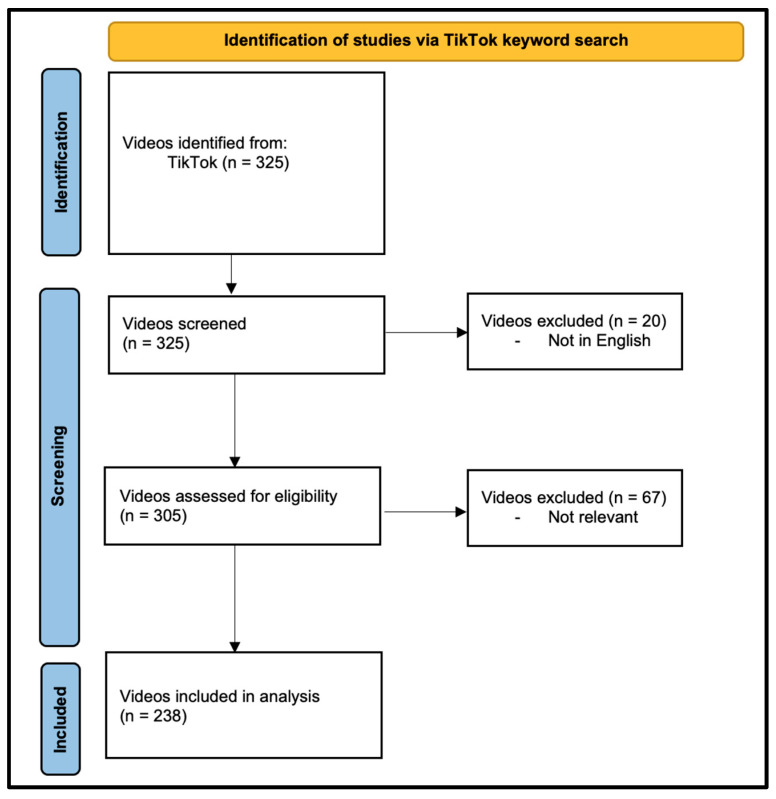
Flowchart of video inclusion criteria.

Of the 325 videos screened, 238 met the inclusion criteria and were analyzed (Figure 1).

As indicated in Table 1, nearly 52% of videos were educational/informational, and 35% included testimonials or were seeking advice. About 60% of videos were created by patients or other individual users, with the majority (92%) being unverified and unsponsored. Over 61% of the videos had an individual in the video, with the remaining (38%) having text or an external voice. Additionally, 81% of videos were created by females, and 73% by white creators based on reviewers’ perceptions. Nearly 60% of video creators did not reveal their qualifications. Of the remaining video creators, 9.7% were physicians and 18.5% were dieticians. In about 51% of videos, the creators had PCOS, 16% discussed stigma attached to PCOS, and 11% reported adverse interactions with their healthcare providers (Table 1).

Table 2 provides the social media engagement statistics of the included videos. The median length of the videos was 42 [Q1–Q3 (15–73)] seconds. The median views, likes, comments, saves, shares, and followers were 468,400; 18,000; 213; 2889; 691; and 477,100, respectively.

As reported in Table 3, the average likes and comments were significantly higher for non-educational videos than for educational videos (*p* = 0.023).

### 3.2. Results of Content Analysis

**Figure 2:**
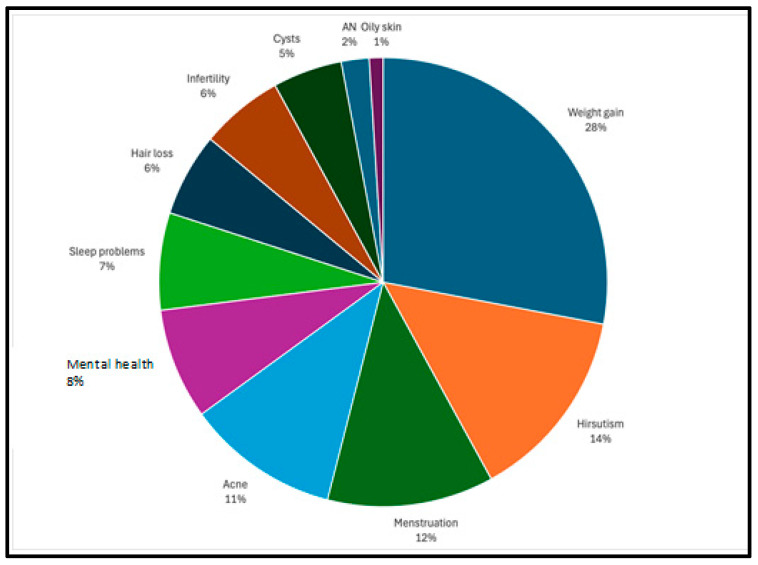
Symptoms discussed in TikTok videos. Videos may have discussed multiple symptoms (n = 489). AN = Acanthosis nigricans.

**Figure 3:**
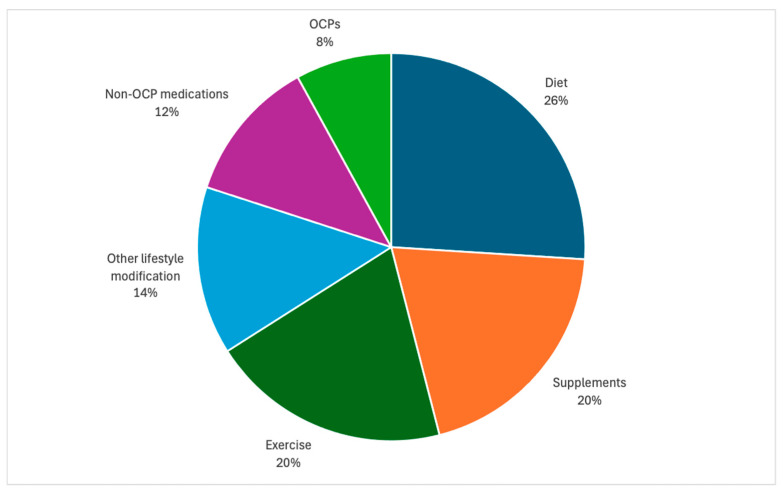
Interventions discussed in TikTok videos. Videos may have discussed multiple interventions (n = 337).

Most videos referenced multiple symptoms associated with PCOS. Within all the videos, 489 individual symptoms were mentioned. The most commonly discussed symptom was weight gain (28%), followed by hirsutism (14%) (Figure 2). When speaking about possible interventions (n = 337), diet was most commonly mentioned (26%). Supplements (20%) and exercise (20%) were also referenced frequently (Figure 3).

### 3.3. Video Content in Misalignment with ACOG Standards

#### 3.3.1. Vitamins and Supplements

Forty-nine (20.6%) videos discussed vitamins or supplements in the management of PCOS. Of those 49 videos, the most commonly discussed supplements included inositol (n = 26), omega-3 (n = 13), magnesium (n = 11), berberine (n = 10), and vitamin D (n = 9).

#### 3.3.2. Diet and Exercise

Thirty-seven (15.5%) videos discussed specific diet or exercise interventions in managing PCOS. Of those 37 videos, the most common dietary recommendations included gluten-free (n = 7), dairy-free (n = 7), and high protein (n = 5) diets. The most commonly discussed exercise regimen was low-intensity exercise/decreased cardio (n = 18).

#### 3.3.3. Teas, Herbs, and Drinks

Twenty-two (9.2%) videos discussed teas, herbs, or other drinks in the management of PCOS. In these 22 videos, spearmint tea (n = 13) and green tea (n = 6) were the most commonly discussed interventions.

#### 3.3.4. Symptoms

Forty-five (18.9%) of videos discussed symptoms not mentioned in the ACOG practice bulletin on PCOS. The most commonly described symptoms included food cravings (n = 10), moon facies (n = 6), and change in libido (n = 5).

A complete list of video content that is misaligned with ACOG guidelines can be found in Appendix A.

### 3.4. Reliability of the Videos

Upon assessing the quality of these videos using the modified DISCERN criteria, no statistically significant differences were found in the mean scores among the two reviewers, indicative of substantial agreement (Table 4). Higher mean scores were seen in educational videos and those posted by physicians, with a statistically significant mean difference (*p* < 0.001), as opposed to non-educational videos and those posted by non-physicians and non-healthcare providers (Table 5).

As reported in Table 6, the odds of sharing educational videos by healthcare professionals were 10.9 times greater than non-healthcare professionals (adjusted odds ratio: 10.9 [95% CI: 5.2–23.1], *p* < 0.001).

## 4. Discussion

Our analysis found that while TikTok videos discussing PCOS received substantial engagement, the overall quality of the information is low, particularly when produced by non-healthcare professionals.

One key finding was that over 65% of videos related to PCOS were created by individuals without healthcare qualifications, including patients, educational platforms, for-profit companies, and other individuals without listed qualifications. The high engagement of these videos, as shown by the millions of views and thousands of likes and comments, demonstrates the broad audiences reached by these creators. Videos created by non-healthcare professionals received more likes on average than those created by physicians and non-physician healthcare professionals.

In categorizing videos based on educational value, videos that were found to be “non-educational” had significantly more likes and views than those that were “educational”. Interestingly, a 2022 study published in Proceedings of the National Academy of Sciences of the United States of America, which looked at the influences surrounding content posted on social media, found that video interaction, such as likes and comments, leads to habit formation among users [17]. They found that once the habit of increased engagement of their videos is formed, users continue their behaviors without considering the outcomes of their actions, such as spreading misinformation. Their hypothesis that false news is part of learned behavior could explain our finding that the less educational videos had the highest engagement. Additionally, it has been hypothesized that content spreading misinformation or evoking strong emotions such as anger or opposition receives more attention and is more likely to go viral [18,19]. Additionally, short-form videos, typically lasting from a few seconds to a few minutes, have seen a surge in popularity due to their concise format and lower production costs, which contribute to increased engagement and more positive viewer responses [20,21,22]. Furthermore, image-based posts, compared to text-only content, have also been shown to generate more engagement [23]. In our study, the average video length was under one minute, which may account for the high levels of likes, comments, saves, and views. While our study did not specifically compare engagement between “educational” and “non-educational” videos or examine the influence of text versus video presenters, these are important areas for future research to better inform healthcare professionals seeking to leverage social media for patient education.

These “non-educational” videos received significantly lower modified DISCERN scores (*p* < 0.001), indicating that the information they provide is often unreliable. This finding suggests that TikTok users are likely exposed to a vast amount of misinformation, which could influence their understanding of PCOS and available therapies.

Conversely, videos produced by physicians and other healthcare professionals, though fewer in quantity overall, were associated with significantly higher DISCERN scores and had a significantly higher likelihood of being “educational”, reflecting higher quality information. These videos garnered less engagement than those made by non-healthcare professionals. This may reflect the broader trend of social media favoring engaging and easily digestible content over scientific and evidence-based information [24]. These trends highlight a critical challenge: while healthcare professionals are more likely to create reliable content, their influence may be limited by what is popularized by audiences on social media platforms.

Our findings regarding the poor quality of information on social media align closely with those reported in the current literature. The concept of “fake news” gained prominence around 2016, particularly during the U.S. presidential election [25]. Since then, the spread of false information has become an increasing concern. Research indicates that fake news is particularly harmful because it is designed to mimic key features of credible news—such as accuracy, verifiability, conciseness, balance, and truthfulness—to deceive the public [26,27]. Recent studies have highlighted the dangers of misinformation not only in the political realm but across multiple sectors, including business, consumer reviews, climate change discussions, popular culture, and healthcare [25,28,29,30]. Our findings further underscore the growing societal impact of misinformation on social media, contributing to issues such as public mistrust and psychological distress [31].

Our findings also highlight the concerning nature of interactions with healthcare providers discussed in the videos. A notable proportion of users who mentioned an interaction with a physician described it as negative, which may contribute to the dissatisfaction many women report with their PCOS diagnosis and treatment options. This dissatisfaction with providers, noted in other studies, may also drive patients to seek information from less reliable sources on TikTok, perpetuating the cycle of misinformation [32,33]. The prevalence of negative interactions with healthcare providers signals the need for improved communication and support within clinical settings to address the concerns of patients with PCOS.

In terms of content, symptoms and interventions discussed were compared with those discussed in the practice bulletin on polycystic ovary syndrome produced by the ACOG [13]. This piece of literature, created by the governing body of obstetrics and gynecology, is considered the standard of care by most practitioners in the field. The most frequently discussed symptoms in the videos were weight gain and hirsutism, which are common symptoms of PCOS discussed in the practice bulletin. However, the ACOG does not formally endorse several other symptoms discussed, such as food cravings and moon facies. Moreover, the practice bulletin states that “there is no ideal dietary modification for women with PCOS beyond caloric restriction”, yet the TikToks analyzed were filled with specific food recommendations and restrictive diets [13]. Additionally, supplements are never discussed within the ACOG bulletin, yet an astounding 20.6% of videos recommended a supplement, and 9.2% recommended a tea or other herb. While some of the interventions mentioned are commonly recommended for managing PCOS symptoms, how they are presented in the videos, with limited or no reference to the scientific literature, raises concerns about the potential for misinformation [34,35]. Videos created by healthcare professionals were more likely to provide accurate information and acknowledge areas of uncertainty, which is essential for helping patients understand the complexity of PCOS and the need for individualized treatment [36].

This study brings to light broader implications for healthcare providers. As patients increasingly turn to social media for health information, providers must be made aware of the content their patients are consuming and the potential misconceptions they may have. A 2021 study underscored the “3 Rs of Social Media”, including reviewing, recognizing, and responding to public health content shared on social media. It was emphasized that the target population should be reviewed, their needs recognized, and their responses should be tailored to disseminate healthcare education to the target audience [37]. Thus, healthcare professionals should consider leveraging social media platforms to provide accurate and evidence-based information in a format that reaches broad audiences to increase access to reliable health education.

### Strengths and Limitations

The strengths of this study include its size and systematic search method. It provides a combination of content analysis and assessment of video quality. This allows providers to understand the possible misinformation circulating on TikTok and provides solutions for providers.

The limitations of this study include the following. First, the inability to ascertain specific characteristics of videos, including the country of origin. Additionally, sampling bias as the content of videos may not be posted by the broader population of individuals with PCOS. Next, the algorithm influence might have influenced which videos are seen and how frequently. In other words, videos that align with the platform’s algorithmic preferences might be over-represented. Additionally, TikTok’s ‘search’ function may not represent what individual users see on their For You page. As TikTok content is constantly changing, the search may yield different videos depending on the timeframe during which they are viewed, which is certainly a temporal limitation. Next, video length, format, and quality varied, making comparisons challenging. Lastly, this study might not account for user interactions outside the video content itself, such as discussions in comments or additional user-generated content that could provide a complete picture of PCOS-related discourse.

## 5. Conclusions

As social media platforms, such as TikTok, continue to grow in popularity, particularly among adolescents and young adults, the availability of unregulated information can become dangerous. The content is predominantly created by non-professional individuals, who have the highest levels of engagement but the lowest reliability scores. These videos often endorsed unproven interventions and discussed negative interactions with physicians. Understanding the PCOS content available to audiences will allow providers to address misconceptions better and provide more focused and informative counseling. Providers may utilize TikTok to create their own videos, thereby positively influencing the platform’s content.

### Research Implications

Ultimately, while TikTok serves as a platform for raising awareness and fostering community among individuals with PCOS, the overall quality of information falls far below that of the high-quality, peer-reviewed literature. Future studies should explore strategies to enhance the visibility and engagement of content produced by healthcare professionals on TikTok and other social media platforms. Additionally, further research must be undertaken to understand the impact of medical misinformation on social media to improve patient outcomes in patients with PCOS and other chronic conditions. As a variety of factors, including culture, age, and other demographic variables can influence user engagement and content preferences, future studies should be conducted to analyze the implications of these differences and help tailor more effective communications to these groups.

## Figures and Tables

**Table 1:** Characteristics of TikTok videos and video creators (n = 238).

Variable	Categories	n (%)	95% CI of Proportion (LCL, UCL)
Characteristics of videos			
Video category	Educational/informational	124 (52.1%)	(45.6, 58.6)
	Testimonial and seeking advice	85 (35.7%)	(29.6, 42.2)
	Advertisement	10 (4.2%)	(2, 7.6)
	Humor/entertainment	19 (8%)	(4.9, 12.2)
Video source (as reported by the video creator)	Physician	23 (9.7%)	(6.2, 14.2)
	Non-physician healthcare provider	52 (21.8%)	(16.8, 27.6)
	Patient or other individual user	141 (59.2%)	(52.7, 65.6)
	Other (student, talk show, educational platform, for-profit company, other organization)	22 (9.2%)	(5.9, 13.7)
Verified	Yes	19 (8%)	(4.9, 12.2)
	No	219 (92%)	(87.8, 95.1)
Sponsored	Yes	17 (7.1%)	(4.2, 11.2)
	No	221 (92.9%)	(88.8, 95.8)
Mode of delivery in the video	Individual in video	146 (61.3%)	(54.8, 67.6)
	Text or external voice	92 (38.2%)	(32.0. 44.7)
Characteristics of the video creator(s) as perceived by the reviewer			
Sex	Female	192 (80.7%)	(75.1, 85.5)
	Male	35 (14.7%)	(10.5, 19.9)
	Unable to determine	11 (4.6%)	(2.3, 8.1)
Race	White	173 (72.7%)	(66.6, 78.3)
	Black	31 (13%)	(9, 18)
	Asian	17 (7.1%)	(4.2, 11.2)
	Unable to determine	17 (7.1%)	(4.2, 11.2)
Age	Young adult	140 (58.8%)	(52.3, 65.1)
	Adult	71 (29.8%)	(24.1, 36.1)
	Older adult	10 (4.2%)	(2, 7.6)
	Unable to determine	16 (6.7%)	(3.9, 10.7)
Qualification of the video creator as reported by creator(s)			
Qualification	Physician	23 (9.7%)	(6, 2, 14.2)
	Other healthcare	15 (6.3%)	(3.6, 10.2)
	Other health educator	11 (4.6%)	(2.3, 8.1)
	Dietician	44 (18.5%)	(13.8, 24)
	Nurse	1 (0.4%)	(0.01, 2.3)
	Student (medical student, other health professions student)	2 (0.8%)	(0.1, 3)
	Teacher	1 (0.4%)	(0.01, 2.3)
	Not available	141 (59.2%)	(52.7, 65.6)
Characteristics discussed in videos			
Mentions having PCOS	Yes	121 (50.8%)	(44.3, 57.4)
	No	117 (49.2%)	(42.6, 55.7)
Stigma of PCOS	Yes	37 (15.5%)	(11.2, 20.8)
	No	201 (84.5%)	(79.2, 88.8)
Interaction with provider	Yes-positive	4 (1.7%)	(0.5, 4.3)
	Yes-neutral	7 (2.9%)	(1.2, 6)
	Yes-negative	25 (10.5%)	(6.9, 15.1)
	No	202 (84.9%)	(79.7, 89.2)

CI: confidence interval; LCL: lower confidence limit; UCL: upper confidence limit.

**Table 2:** Descriptive statistics of social media engagement of included videos (n = 238).

Video Characteristics	Minimum	Maximum	Mean (SD)	95% CI of Mean	Median (Q1; Q3)
Days since upload	4	1478	388.7 (291.7)	(350.7; 425.5)	331.00 (174; 525.5)
Video length in seconds	5	492	58.9 (63.0)	(51; 67.2)	42.00 (15; 73)
Views	292	85,100,000	1,672,362.2 (6,305,197.8)	(870,956.1; 2,487,877.7)	468,400.00 (146,400; 1,100,000)
Likes	0	3,500,000	100,647.8 (360,609.5)	(54,835.4; 147,309.5)	18,000.00 (5631; 65,100)
Comments	0	30,000	759.9 (2556.8)	(435.3; 1090.9)	213.00 (56; 668.5)
Saves	0	425,900	16,312.4 (47228.5)	(10,326.2; 22,436.3)	2889.00 (840; 10,150)
Shares	0	248,200	4681.4 (19,539.6)	(2195.7; 7206.6)	691.00 (191; 2370.5)
Views/day	0.5	1810,638.3	15,410.3 (119,416.1)	(161.9; 30,788.8)	1431.7 (398.2; 5672.4)
Views/like	6.4	167.5	29.8 (23.5)	(26.8; 32.8)	23.3 (14.8; 36.0)
Likes/day	0.000	74468.1	779.01 (5130.7)	(124.4; 1440.2)	62.2 (16.1; 256.7)
Followers	86	53,00,000	581,027 (644,249.4)	(501,001.6; 665,954.4)	477,100.00 (80,950; 977,700)

**Table 3:** Video engagement metrics, including views, likes, and comments, comparing video categories and user qualifications.

Variable	Categories	Likes (Mean ± SD)	*p* Value	Views (Mean ± SD)	*p* Value	Comments (Mean ± SD)	*p* Value
Video type	Educational	49,870.8 ± 174,694.4	0.023	962,717.9 ± 199,7483.1	0.070	399.5 ± 553.6	0.023
	Non-educational	155,878.8 ± 483,333.7		2,444,256.1 ± 8,825,208.4		1151.9 ± 3616.6	
Qualification	Physician	15,873.1 ± 2156.7	0.175	393,107.4 ± 526,680	0.337	313.6 ± 529.5	0.233
	Non-physician healthcare provider	46,882.5 ± 60,561.5		998,841.5 ± 1,218,247.5		356 ± 373.3	
	Non-healthcare professionals	129,762 ± 431,536.3		2067,736.1 ± 7,557,825		951.7 ± 3060.1	

*p* values less than 0.05 are considered statistically significant.

**Table 4:** Comparing mean scores of modified DISCERN criteria between reviewer A and reviewer B.

Variable	Reviewer A	Reviewer B	*p* Value
Modified DISCERN	1.7 ± 1.2	1.8 ± 1.2	0.381

**Table 5:** Modified DISCERN scores for educational versus non-educational videos and videos published by healthcare professionals versus non-healthcare professionals.

Variable	Categories	Modified DISCERN (mean ± SD)	*p* Value
Video type	Educational/informational	2.4 ± 1.2	<0.001
	Non-educational (including testimonial, advertisement, humor/entertainment)	1.1 ± 0.6	
Source	Physician	3.6 ± 0.9	<0.001
	Non-physician healthcare provider	2.0 ± 1.1	
	Non-healthcare individuals (patient, other individual user, student, talk show, educational platform, for-profit company, other organization)	1.0 ± 0.2	

*p* values less than 0.05 are considered statistically significant.

**Table 6:** Logistic regression predicting the likelihood of videos shared by healthcare professionals vs. non-healthcare professionals.

Variable	Odds Ratio	95% CI: LCL, UCL	*p* Value
Video type			
Educational (ref: non-educational)	10.9	(5.2, 23.1)	<0.001
Mentions stigma of PCOS			
Yes (ref: no)	0.7	(25.5, 206.1)	0.547

*p* values less than 0.05 are considered statistically significant.

## Data Availability

No new data were created or analyzed in this study.

## Appendix A

Symptoms and interventions discussed in TikTok videos not in alignment with ACOG guidelines

**Vitamins and Supplements**	**# of Videos**
Inositol/Ovasitol	26
Omega-3/fish oil	13
Magnesium	11
Bernerine	10
Vitamin D	9
NAC	7
Zinc	7
Probiotic	7
L-Carnitine	6
Multivitamin	6
CoQ10	5
Vitamin B	4
Saw palmetto	4
Ashwaganda	4
Other *	18
* Vitamin C (n = 2), curcumin (n = 2), maca root (n = 2), sea moss (n = 2), chromium (n = 1), biotin (n = 1), calcium (n = 1), chlorophyll drops (n = 1), iron tonic (n = 1), womb tonic (n = 1), collagen (n = 1), methyconolamine (n = 1), L-glutamine (n = 1), Mucinex (n = 1).

**Teas, Herbs, and Other Drinks**	**# of Videos**
Spearmint tea	13
Green tea	6
Turmeric	4
Other *	15
* Ginger (n = 3), dandelion (n = 2), cinnamon (n = 2), apple cider vinegar (n = 2), adrenal cocktail (n = 2), elderberry (n = 1), pomegranate (n = 1), peppermint (n = 1), turmeric (n = 1).

**Diet and Exercise**	**# of Videos**
Low-intensity workouts/decrease cardio	18
Gluten-free diet	7
Dairy-free diet	7
High-protein diet	5
Eliminate processed foods	4
Decrease caffeine	4
Anti-inflammatory foods	4
Other *	10
* Avoid alcohol (n = 2), increase fiber (n = 1), alkaline foods (n = 1), low glycemic index (n = 1), Mediterranean diet (n = 1), specific food recommendation (n = 4).

**Symptoms**	**# of Videos**
Cravings	10
Moon facies	6
Decreased sex drive	3
Increased sex drive	2
Commitment issues	2
Seborrheic dermatitis	2
Bloating	2
Brain fog	2
Other *	16
* Vaginal dryness (n = 1), scleral icterus (n = 1), facial flushing (n = 1), red palms (n = 1), facial swelling (n = 1), temperature sensitivity (n = 1), neck hump (n = 1), build muscle (n = 1), purple stretch marks (n = 1), hyperpigmentation (n = 1), headaches (n = 1), decreased breast size (n = 1), hair follicles on ovaries (n = 1), gut issues (n = 1), hypoglycemia episodes (n = 1), constipation (n = 1).
